# A therapy with miglustat, 2-hydroxypropyl-ß-cyclodextrin and allopregnanolone restores splenic cholesterol homeostasis in Niemann-pick disease type C1

**DOI:** 10.1186/s12944-019-1088-2

**Published:** 2019-06-28

**Authors:** Anna-Maria Neßlauer, Anne Gläser, Markus Gräler, Robby Engelmann, Brigitte Müller-Hilke, Marcus Frank, Christine Burstein, Arndt Rolfs, John Neidhardt, Andreas Wree, Martin Witt, Anja U. Bräuer

**Affiliations:** 1 Institute of Anatomy, Rostock University Medical Center, Gertrudenstraße 9, 18057 Rostock, Germany; 2 Research Group Anatomy, School of Medicine and Health Sciences, Department für Humanmedizin, Abteilung Anatomie, Carl von Ossietzky University Oldenburg, Carl-von-Ossietzky Straße 9-11, 26129 Oldenburg, Germany; 3 Department of Anesthesiology and Intensive Care Medicine, Center for Sepsis Control and Care (CSCC), and the Center for Molecular Biomedicine (CMB), Jena University Hospital, Hans-Knöll-Str. 2, 07745 Jena, Germany; 4 Institute of Immunology, Rostock University Medical Center, Schillingallee 70, 18057 Rostock, Germany; 5 Medical Biology and Electron Microscopy Center, Rostock University Medical Center, Strempelstraße 14, 18057 Rostock, Germany; 6 Institute of Clinical Chemistry and Pathobiochemistry, Rostock University Medical Center, Ernst-Heydemann-Straße 6, 18057 Rostock, Germany; 7 Centogene AG, Am Strande 7, 18055 Rostock, Germany; 8 Human Genetics, Faculty of Medicine and Health Sciences, University of Oldenburg, Oldenburg, Germany; 9 Research Center for Neurosensory Science, Carl von Ossietzky University Oldenburg, Oldenburg, Germany

**Keywords:** Niemann-pick disease type C1, Spleen, Phospholipids, PRGs, G-protein-coupling receptor, qRT-PCR, S1P, HPTLC, Lymphocyte

## Abstract

**Background:**

Niemann-Pick disease type C1 (NPC1) is an autosomal-recessive lipid-storage disorder with an estimated minimal incidence of 1/120,000 live births. Besides other neuronal and visceral symptoms, NPC1 patients develop spleen dysfunction, isolated spleno- or hepatosplenomegaly and infections. The mechanisms of splenomegaly and alterations of lipid metabolism-related genes in NPC1 disease are still poorly understood.

**Methods:**

Here, we used an NPC1 mouse model to study a splenoprotective effect of a treatment with miglustat, 2-hydroxypropyl-ß-cyclodextrin and allopregnanolone and showed that this treatment has a positive effect on spleen morphology and lipid metabolism.

**Results:**

Disease progress can be halted and blocked at the molecular level. Mutant *Npc1* (*Npc1*^*−/−*^) mice showed increased spleen weight and increased lipid accumulation that could be avoided by our treatment. Also, FACS analyses showed that the increased number of splenic myeloid cells in *Npc1*^*−/−*^ mice was normalized by the treatment. Treated *Npc1*^*−/−*^ mice showed decreased numbers of cytotoxic T cells and increased numbers of T helper cells.

**Conclusions:**

In summary, the treatment promotes normal spleen morphology, stabilization of lipid homeostasis and blocking of inflammation, but alters the composition of T cell subtypes.

**Electronic supplementary material:**

The online version of this article (10.1186/s12944-019-1088-2) contains supplementary material, which is available to authorized users.

## Background

Niemann-Pick Disease Type C1 (NPC1) is a rare, autosomal-recessive, lysosomal lipid storage disease with hepatosplenomegaly and progressive neurological involvement [1–4]. The fatal disease progresses rapidly and finally ends with a substantial loss of physical and mental abilities [5]. An NPC1 mutation is responsible for 95% of NPC patients [6]. A loss-of-function NPC1 protein leads to abnormal intracellular trafficking of lipids [7–9]. Normally, the luminal NPC2 and transmembrane NPC1 proteins trap unesterified cholesterol and transfer lipids out of late endosomes/lysosomes (LE/LY) [9, 10]. The malfunction of the NPC1 protein results in a toxic accumulation of unesterified cholesterol, sphingosine, sphingomyelin, glycolipids, glycosphingolipids (GSLs), and fatty acids, most likely as a consequence of impaired activity of multiple lysosomal hydrolases [4, 11, 12]. Altered lipid metabolism has been found in many tissues and organs, including brain, liver and spleen [13]. Moreover, it is associated with the infiltration of lipid-overloaded macrophages (foam cells) into many organs, leading to parenchymal cell death [14]. One of the hallmarks of this disease, i.e. the enlarged and severely disrupted morphological structures in the spleen, have been identified in NPC1 patients, being often the first sign of NPC1 disease before neurological symptoms appear [15]. The *BALB/cNctr-Npc1*^*m1N*^*/J* mouse model partly mimics the human disease, resulting in neurovisceral lipid storage and progressive neurodegeneration [1, 16, 17].

In addition to other tasks, the spleen is responsible for clearance of red blood cells and immune defense [18]. The mutant *Npc1* (*Npc1*^*−/−*^) mice show immune dysfunction, which is seen in a modified distribution and function of natural killer cells (NK cells) [19, 20]. NK cells play a role in killing of virally infected and transformed cells [21]. It has been shown that the altered NK cell frequency in *Npc1*^*−/−*^ mice is similar to the decrease in NK cell frequency in the blood of NPC1 patients, with an important clinical relevance [20]. Furthermore, the metabolism of the bioactive lipid sphingosine-1-phosphate (S1P) and the S1P receptor activities contribute to many regulatory processes in the immune system [22, 23]. Normally, S1P is exported out of the cell. Extracellular S1P acts as a ligand of five G-protein-coupled receptors called sphingosine-1-phosphate receptors 1–5 (S1PR1–5). S1P modulates, depending on the S1P receptor to which it is coupled, multiple signal transduction pathways, inducing proliferation, apoptosis, and motility [22, 23]. The involvement of NPC1 in sphingosine efflux of the lysosomes leads to reduced cellular S1P level in *Npc1*^*−/−*^ mice and, therefore, alters these pathways [20, 24].

So far, disease-specific therapy options are limited. In the absence of a causal therapy, the substrate miglustat (Zavesca®, Actelion Pharmaceuticals, Allschwil, Switzerland) is the only approved drug for the treatment of progressive neurological manifestations of NPC1 disease in Europe [25]. This small iminosugar reversibly inhibits glucosylceramide synthase and is a key component of glycosphingolipid biosynthesis [26]. The therapeutic potential of miglustat in stabilizing or slowing disease progression has been confirmed in numerous clinical trials [27, 28]. Furthermore, additional studies have demonstrated the positive effect of the cyclic oligosaccharide 2-hydroxypropyl-ß-cyclodextrin (HPßCD) in reducing the lysosomal cholesterol accumulation seen in the prolonged lifespan and delayed neurodegeneration in *Npc1*^*−/−*^ mice [28]. To find an improved therapeutic approach to the treatment of NPC1, we applied in this paper a therapy of miglustat with HPßCD and allopregnanolone [29]. Recent studies in *Npc1*^−/−^ mice showed that this therapy reduces intracellular lipid accumulation in numerous organs, among others in the liver. Moreover, therapy decreased cerebellar neurodegeneration by significantly prolonging survival of Purkinje cells, improved sensory perception by increased regeneration of the olfactory epithelium, and reduced motor deficits [1, 30–32]. Taken together, the therapy delays the onset and inhibits the progression of the disease, and prolongs life expectancy.

To better understand the mechanism of the pharmacological treatment on special organs in *Npc1*^*−/−*^ mice, we here studied the spleen with respect to morphology, lipid metabolism, and the effects at the cellular level. Morphological characteristics of spleen tissue were assessed by histology, immunohistochemistry and transmission electron microscopy (TEM). Biochemical parameters were investigated with high-performance thin-layer chromatography (HPTLC), mass spectrometry, and quantitative real-time PCR (qRT-PCR). Effects at the cellular level were registered using fluorescence-activated cell sorting (FACS) and blood count analysis.

## Material and methods

### Animals

Heterozygous breeding pairs of *BALB/cNctr-Npc1*^*m1N*^*/J* (*Npc1*^*−/−*^) mice were obtained from the Jackson Laboratories (Bar Harbor, ME, USA) for generating homozygous *Npc1*^*−/−*^ mutants and control wild type (*Npc1*^*+/+*^) mice. In accordance with German and European guidelines (2010/63/EU) for the use of laboratory animals, mice were kept under standard laboratory conditions (12 h light/dark cycle; 55 ± 15% humidity; 24 ± 2 °C room temperature, and water and food ad libitum). Approval of experiments was obtained from the local ethics body of the state of Mecklenburg Vorpommern (approval IDs: LALLF M-V/ TST/7221.3–1.1-011/16 and LALLF M-V/ TST/7221.3–1.1-030/12).

Genotyping was conducted until postnatal day 7 (P7) by PCR analysis. Sham-treated *Npc1*^*+/+*^ (*n* = 11) and sham-treated *Npc1*^*−/−*^ (*n* = 9) mice, which received normal saline solution or Ringer’s solution without active substances, were evaluated. Additionally, *Npc1*^*+/+*^ mice (*n* = 14) and *Npc1*^*−/−*^ mice (*n* = 12) that received a treatment were examined.

### Genotyping

For genotyping by PCR analysis, 1–2 mm of the tails were clipped at P6 and homogenized in DirectPCR-Tail and 1% proteinase K (Peqlab, Erlangen, Germany) at 55 °C with 750 rpm for 16 h overnight on a Thermo Mixer (Eppendorf, Hamburg, Germany). Extracts were centrifuged for 30 s with 6000 rpm and PCR analyses were performed twice with 2 μl of the lysate and two different primer pairs under equal cycling conditions. For detecting the mutant allele (obtained fragment size 475 bp), primers 5′-ggtgctggacagccaagta-3′ and 5′-tgagcccaagcataactt-3′, and for the wild type allele (obtained fragment size 173 bp) 5′-tctcacagccacaagcttcc-3′ and 5′-ctgtagctcatctgccatcg-3′ were used.

### Pharmacological treatment

Four groups were systematically evaluated: sham-treated *Npc1*^*+/+*^ mice, sham-treated *Npc1*^*−/−*^ mice, treated *Npc1*^*+/+*^ and treated *Npc1*^*−/−*^ animals. The treatment scheme was as described previously [30, 32].

**Fig. 1:**
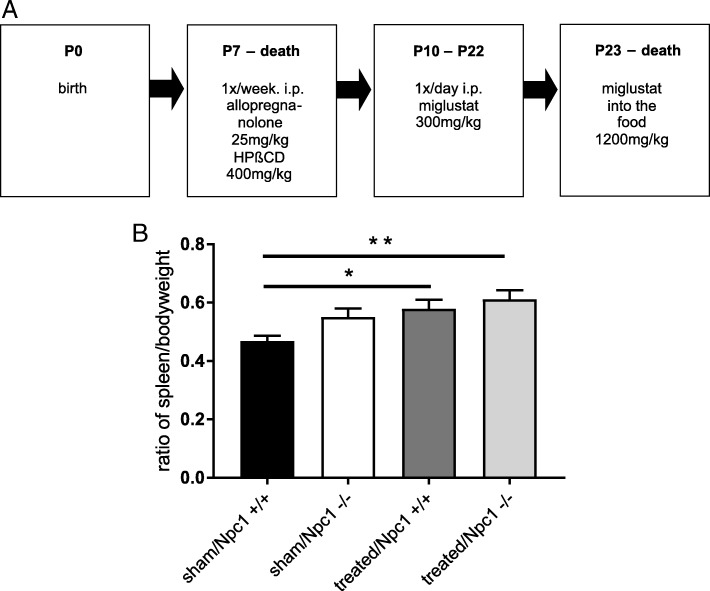
Scheme of the drug application for the treatment in *Npc1*^*+/*+^ and *Npc1*^*−/−*^ mice (**a**). Evaluation of spleen-to-body-weight ratios (SW/BW) of sham-treated *Npc1*^*+/+*^ (*n* = 11), sham-treated *Npc1*^*−/−*^ (*n* = 9), treated *Npc1*^*+/+*^ (*n* = 14), and treated *Npc1*^*−/−*^ mice (*n* = 12, **b**). Note the increase of SW/BW ratio of treated *Npc1*^*+/+*^ and treated *Npc1*^*−/−*^ mice. Values are given as mean ± SEM; ANOVA; multiple comparison tests: *p ≤ 0.05 sham-treated *Npc1*^*+/+*^ vs. treated *Npc1*^*+/+*^; **p ≤ 0.01 sham-treated *Npc1*^*+/+*^ vs. treated *Npc1*^*−/−*^

The combination treatment (in the following referred to as “treated”), starting at P7, includes weekly injection of mice with 2-hydroxypropyl-ß-cyclodextrin (HPßCD, 4000 mg/kg, i. p., Sigma Aldrich, St. Louis, MO, United States) and allopregnanolone (Pregnan-3alpha-ol-20-one; Sigma Aldrich, St. Louis, MO, United States) (25 mg/kg allopregnanolone dissolved in 40% HPßCD in Ringer’s solution). Additionally, from P10 to P22 mice were injected daily with miglustat (N-butyldeoxynojirimycin, Zavesca®; Actelion Pharmaceuticals, Allschwil, Switzerland), dissolved in 0.9% NaCl solution, 300 mg/kg i. p.). Thereafter, miglustat powder was mixed with standard chow and administered until P65, resulting in a daily intake of 1200 mg/kg miglustat. “Sham-treated” *Npc1*^*+/+*^ and *Npc1*^*−/−*^ mice were injected with Ringer’s solution or normal saline solution following the same treatment scheme (Fig. 1a). Animals were sacrificed at P65.

### Sampling and assays

All mice were deeply anesthetized with pentobarbital (90 mg/kg, AbbVie, Berlin, Germany), weighed and exsanguinated by puncture of the vena cava inferior for immediate separation of plasma, followed by harvest of non-perfused spleen tissue. The spleens were weighed, photographed and subsequently divided into 3 parts. The first part was frozen in liquid nitrogen and stored at − 80 °C for quantitative real-time-PCR analysis (qRT-PCR), high-performance thin-layer chromatography (HPTLC) analysis, and mass spectrometry. The second part was fixed in 4% paraformaldehyde (PFA) for histology and immunohistochemistry. The third was stored in DMEM for direct-flow cytometry analysis and cell sorting.

### Blood cell analysis

EDTA samples of 200 μl of whole blood were drawn from the vena cava inferior to analyse leukocyte count (WBC = white blood cells), erythrocyte count (RBC = red blood cells), hematocrit, hemoglobin, platelet count (PLT = platelets), and leukogram counts for neutrophil granulocytes (neutrophils), basophil granulocytes (basophils), lymphocytes, and monocytes. Blood cell counting (WBC, RBC, PLT) and WBC differential counts were performed using the Sysmex XE-5000 (Sysmex Austria, Vienna, Austria) automated hematology system. The Sysmex XE-5000 utilized impedance technology for RBC and PLT counts. For the WBC count and differential, the system utilized data from impedance, light scattering, and fluorescence measurements. The photometric measurement of hemoglobin concentrations on the XE-5000 employed the sodium-lauryl sulfate (SLS) method.

### Flow cytometry analysis and cell isolation (FACS)

Spleens were homogenized using a stainless-steel mesh screen and a 70 μm cell strainer. Subsequently, erythrocytes were lysed with a solution containing 155 mM NH_4_Cl, 10 mM KHCO_3_ and 0.1 mM EDTA for 5 min on ice. Splenocytes were analysed by flow cytometry using a lymphocyte (CD4:FITC [clone GK1.5], CD8:PE [clone 53–6.7], B220:PE-Cy7 [clone RA3-6B2], and CD3:APC [clone 145-2C11]), and a myeloid cell panel (CD11b:PE [clone M1/70], F4/80:APC [clone BM8], CD11c:Alexa488 [clone N418], and CD169:PE-Cy7 [clone 3D6.112]). All antibodies were bought from BioLegend, San Diego, CA, USA.

B cells were isolated from splenocyte suspension using B220 Microbeads (Miltenyi Biotec, Bergisch Gladbach, Germany) according to the manufacturer’s protocols. T cells were isolated from the unlabeled cell fraction after B cell isolation using the Pan T Cell Isolation Kit II (Miltenyi Biotec, Bergisch Gladbach, Germany) according to the manufacturer’s protocols. The isolation procedures were all performed using a QuadroMACS magnet and LS magnetic columns (Miltenyi Biotec, Bergisch Gladbach, Germany). Isolated cells were frozen and stored at − 80 °C until downstream analyses.

### Mass spectrometry (MS)

Measurements were performed according to protocol using liquid chromatography coupled to triple-quadrupole mass spectrometry as previously described [33]. Tissue samples were homogenized using the Stomacher Model 80 MicroBiomaster Blender (Seward, Worthing, UK) in 5 ml PBS after addition of C17-base sphingosine (Sph), sphingosine-1-phosphate (S1P), lysophosphatidylcholine (LPC), sphingomyeline (SM), phosphatidylcholine (PC 34:0), and C15-base ceramide (Cer) as internal standards (300 pmol/sample, C17-S1P 100 pmol/sample, Avanti Polar Lipids, Alabaster, AL, USA). Supernatants (1 ml) were transferred into glass centrifuge tubes (VWR International, Radnor, PA, USA), mixed with 200 μl hydrochloric acid (6 N; Carl Roth GmbH, Karlsruhe, Germany) and 1 ml methanol (VWR International, Radnor, PA, USA), and vigorously vortexed for 5 min in the presence of 2 ml chloroform (Carl Roth GmbH, Karlsruhe, Germany). Aqueous and chloroform phases were separated by centrifugation for 3 min at 1900 x g, and the lower chloroform phase was transferred into a new glass centrifuge tube. After a second round of lipid extraction with additional 2 ml chloroform, the two chloroform phases were combined and vacuum-dried at 50 °C for 50 min using a vacuum concentrator (RVC 2–25 CD plus, Martin Christ Gefriertrocknungsanlagen GmbH, Osterode, Germany). The extracted lipids were dissolved in 100 μl methanol/chloroform (4:1, v/v) and stored at − 20 °C. Detection was performed with the QTrap triple-quadrupole mass spectrometer (Sciex, Ontario, Canada) interfaced with the 1100 series chromatograph (Agilent Technologies, Waldbronn, Germany) and the Hitachi Elite LaChrom column oven and autosampler (VWR International, Radnor, PA, USA). Positive electrospray ionization (ESI) LC/MS/MS analysis was used for detection of dihydro (DH)-Sph, Sph, S1P, SM, PC, LPC, and C16-Cer. Multiple reaction monitoring (MRM) transitions were as follows: C17-Sph m/z 286/268, DH-Sph m/z 302/284, Sph m/z 300/286, C17-S1P m/z 366/250, S1P m/z 380/264, C15-Cer m/z 524/264, C16-Cer m/z 538/264, SM (17:0) m/z 717/184, SM (16:0) m/z 703/184, PC (34:0) m/z 762/184, PC (34:2) m/z 758/184, LPC (17:0) m/z 510/184, LPC (16:0) m/z 496/184. In addition, MRM transitions (m/z) of the following unknown molecules potentially belonging to the SM and/or PC family were measured: 705/184, 719/184, 781/184, 783/184, 799/184, 801/184, 803/184, 813/184, 831/184, 865/184, 867/184, 883/184, and 885/184. These transitions were identified in a precursor ion scan to provide the highest differences between *Npc1*^*+/+*^ and *Npc1*^*−/−*^ mouse spleens. Liquid chromatographic resolution of all analytes was achieved using a 2 × 60 mm MultoHigh C18 reversed phase column with 3 μm particle size (CS-Chromatographie Service, Langerwehe, Germany). The column was equilibrated with 10% methanol and 90% of 1% formic acid in H_2_O for 5 min, followed by sample injection, and 15 min elution with 100% methanol with a flow rate of 300 μl/min. Standard curves were generated by adding increasing concentrations of the analytes to 300 pmol (100 pmol C17-S1P) of the internal standard. Linearity of the standard curves and correlation coefficients were obtained by linear regression analyses. Data analyses were performed using Analyst 1.6 (Sciex, Ontario, Canada).

### Transmission electron microscopy (TEM)

The three animals of sham-treated *Npc1*^*+/+*^, *Npc1*^*−/−*^ and treated *Npc1*^*+/+*^, *Npc1*^*−/−*^ mice were sacrificed by an overdose of pentobarbital, followed by dissection of the spleens. After preparation, spleen samples were postfixed in 0.1 M phosphate buffer containing 2.5% glutaraldehyde for at least 24 h at 4 °C. Thereafter, the specimens were osmicated, washed, block contrasted with 2% aqueous uranyl acetate, dehydrated through a graded series of ethanol, and embedded in Epon 812 (Plano, Marburg, Germany). Ultrathin sections (about 70 nm) were mounted on pioloform-coated slot copper grids and contrasted with uranyl acetate (4 min) followed by lead citrate (2 min). The specimens were examined with a Zeiss EM 902 transmission electron microscope (Zeiss, Oberkochen, Germany) at 80 kV. Photographs were taken using a CCD camera (Proscan, Lagerlechfeld, Germany) and adjusted using Photoshop CS2 software (Adobe Systems, San José, CA, USA). FACS- separated B and T lymphocytes were centrifuged, osmicated, washed, infiltrated with agar and routinely processed for TEM as described above.

### Lipid extraction

For lipid extraction, one part of the sham and treated *Npc1*^*+/+*^ and *Npc1*^*−/−*^ spleen tissues, stored at − 80 °C, was weighed. Lipids were extracted according to Bligh and Dyer [34], with slight modifications. Chloroform (Merck KGaA, Darmstadt, Germany), methanol (Merck KGaA, Darmstadt, Germany) and hydrochloric acid (Merck KGaA, Darmstadt, Germany) were mixed in the ratio 2:4:1 and added to the tissue. Afterwards, 1% butylated hydroxytoluene (SAFC, Carlsbad, CA, USA) in water (Carl Roth GmbH, Karlsruhe, Germany) was added to prevent lipid oxidation. The tissue was subsequently homogenised with an ultra-turrax T10 (IKA, Staufen, Germany). The fluorescent standard TopFluor-PC (10 μl/50 mg, #810281, Avanti Polar Lipids, Alabaster, AL, USA) was added to the tissue, and used to determine the reproducibility of the lipid extraction method and to detect the loss of lipids during extraction [35]. Chloroform was added to the homogenised tissue and vortexed 3 times, with 10 min breaks between. Next, water (Carl Roth GmbH, Karlsruhe, Germany) was added to the tissue and vortexed 3 times, with a 10 min break between, followed by incubation for 30 min and centrifugation at 1260 x g (PRP centrifuge P002, W Medical Systems, Lauenförde, Germany) for 10 min. The now triphasic separation was visible, and the bottom phase containing a mix of chloroform and lipids was transferred into a brown-glass bottle. Finally, the chloroform was evaporated in an N_2_ chamber at 50 °C overnight. The bottles were stored at − 20 °C until use.

Chromatographic standards were used to identify the lipid class of interest in the samples, as well as to verify that the chromatographic process worked. The standards were applied on the silica gel plate (Merck KGaA, Darmstadt, Germany).

### Separation and analysis of lipid classes by high performance thin-layer chromatography (HPTLC)

The stationary phase was 10 × 10 cm silica gel (Merck KGaA, Darmstadt, Germany). For the mobile phases, chloroform (Merck KGaA, Darmstadt, Germany), methanol (Merck KGaA, Darmstadt, Germany), ammonia 32% (VWR Chemicals, Radnor, PA, USA), and water (Carl Roth GmbH, Karlsruhe, Germany) solution was used in the ratio 161:75:5:10; detection with copper-II-sulphate 10% (Merck KGaA, Darmstadt, Germany), phosphoric acid 8% (Carl Roth GmbH, Karlsruhe, Germany), methanol 5% in water, and baking at 120 °C for 60 min [36, 37]. The plate was scanned in a TLC scanner (CAMAG, Wilmington, NC, USA) and Rf value (retardation factor) and intensity (in arbitrary units (AU)) were compared to standards using the VisionCats program 2.4 (CAMAG, Wilmington, NC, USA). Digital data were processed with CorelDRAW 2017 (Corel Corporation, Ottawa, Canada).

### Histology and immunohistochemistry

Spleen tissue was fixed in 4% paraformaldehyde (PFA) in phosphate buffered saline (PBS) for 1 day, embedded in paraffin, sectioned at 4 μm thickness, and mounted on poly-L-lysine coated glass slides. Four μm sections were cut and stained with hematoxylin and eosin (H&E). For the quantification of proliferating cells, every 10th section was subjected to immunohistochemistry. Sections were deparaffinized, rehydrated and pretreated with microwaves in 0.1 M citrate buffer (5 min, 850 W and 5 min, 340 W) followed by incubation with 3% hydrogen peroxide (H_2_O_2_) in 0.1 M PBS to block endogenous peroxidases for 30 min, and 5% normal goat serum (NGS) in PBS for 45 min to block nonspecific binding sites. Subsequently, sections were exposed to the primary antibody against CD68 (1:100, #MCA1957, Bio-Rad, Hercules, USA), Iba-1 (1:2000, #019–19,741, Wako, Osaka, Japan), CD3 (Ready-to-Use, #GA503, DAKO, Carpinteria, CA, USA), and CD45R (1:200, #11–0460-82, eBiosience, San Diego, USA) in 3% NGS/PBS overnight at 4 °C. One section of each slide was used for negative control. After washing in PBS, the sections were sequentially incubated for 1 h with the secondary anti-rat IgG (1:200; Vector, Burlingame, CA, USA) for CD68, and anti-rabbit IgG (1:200; Vector, Burlingame, CA, USA) for Iba-1 and CD3, the streptavidin-biotin-complex (ABC) reagent for 1 h (Vectastain-Elite; Vector, Burlingame, CA, USA), and finally visualized with H_2_O_2_-activated 3,-3,-diaminobenzidine (DAB, Sigma, Munich, Germany) [38]. Sections were subsequently counterstained with hematoxylin, dehydrated, mounted with DePeX and coverslipped. For controls, primary antisera were omitted. In sections with negative controls, no reactivity was observed [31, 39]*.* Images were obtained using a transmitted-light microscope Olympus BX3F (Olympus K.K., Shinjuku, Tokio, Japan) and a Basler acA2440 camera (Basler AG, Ahrensburg, Germany). Digital data was processed with EasyScan Software 2017c-2 (Smart In Media, Cologne, Germany) and CorelDRAW 2017 (Corel Corporation, Ottawa, Canada).

### RNA extraction and cDNA synthesis

For RNA extraction and cDNA synthesis, spleens from 9 homozygous *Npc1*^*−/−*^ and 3 *Npc1*^*+/+*^ control mice of both sexes were dissected at P65. Mice were deeply anesthetized with pentobarbital (90 mg/kg) and then decapitated. The tissues were collected, flash-frozen in liquid nitrogen and stored at − 80 °C. RNA extraction and cDNA synthesis were performed according to Coiro et al. [40], with slight modifications. TRIzol reagent (Thermo Fisher Scientific, Waltham, MA, USA) was used for homogenization of the tissue, followed by RNA extraction according to the manufacturer’s protocol. After precipitation and drying, RNA was resuspended in an aliquot of RNase and DNase-free water quantified by A_260nm_ spectrophotometry (BioSpectrometer basic, Eppendorf, Hamburg, Germany) and stored at − 80 °C. cDNA was synthesized with 5 μg of total RNA using the High-Capacity cDNA Reverse Transcription Kit (Thermo Fisher Scientific, Waltham, MA, USA) according to the manufacturer’s protocol. Control reactions were performed without MultiScribe Reverse Transcriptase. cDNA was stored at − 20 °C. The quality of amplified cDNA was controlled using *β*-*Actin* PCR.

### Quantitative real-time PCR (qRT-PCR)

**Fig. 2:**
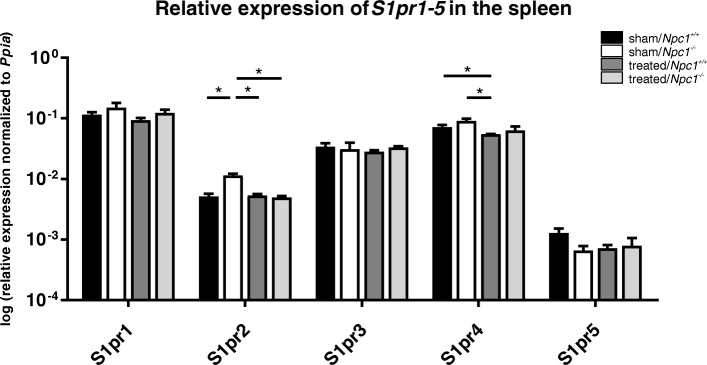
Quantitative RT-PCR of S1pr (Sphingosine-1-phosphate receptor) 1–5 in spleen tissue of sham- and treated *Npc1*^*+/+*^ (sham, *n* = 3; treated, *n* = 3) and *Npc1*^*−/−*^ (sham, *n* = 3; treated, *n* = 3) mice. Sham-treated *Npc1*^*−/−*^ mice demonstrated a noticeable increase of *S1pr2* and *S1pr4*. The expression tended to be normalized after treatment. Data are normalized to Ppia and shown as mean ± SEM. p ≤ 0.05 was considered significant (*p ≤ 0.05). For p-values see text. *S1pr*: Sphingosine-1-phosphat receptor, Ppia: Peptidylprolyl isomerase A

Quantitative RT-PCR followed the protocol of Coiro et al., with slight modifications [40]. Each PCR reaction was performed in duplicate, and contained 8 μl RNase and DNase-free water, 10 μl TaqMan® Universal PCR Master Mix (Thermo Fisher Scientific, Waltham, MA, USA), 1 μl cDNA (0.1 μg/μl), and 1 μl TaqMan Gene Expression Assays for each *S1pr* transcript (Additional file 1: Table S1). mRNA was normalized relative to *cyclophilin A* (*Ppia*) (Fig. 2) [41–43] and *ß-Actin (Actb)* [44], both of which have been proven as useful reference genes for qRT-PCR. PCR thermocycling parameters were: 95 °C for 20 s and 45 cycles of 95 °C for 1 s and 60 °C for 20 s. For analysis of the relative change in gene expression, we used the 2^-ΔCt^ method. The reactions were run on the 7900 HT Fast Real-Time PCR System (Thermo Fisher Scientific, Waltham, MA, USA) using SDS and RQ manager Software 1.2 (Thermo Fisher Scientific, Waltham, MA, USA) or the CFX96 Touch™ Real-Time PCR Detection System (Bio-Rad Laboratories, Hercules, CA, USA) using CFX Manager Software 3.1 (Bio-Rad Laboratories, Hercules, CA, USA). Each value was the average of three separate experiments.

### Statistical analysis

Statistical evaluation of spleen-to-body weight: ratio was carried out with the multiple-comparison test ANOVA using GraphPad Prism 5.0 (GraphPad Software, La Jolla, CA, USA). A two-tailed, non-parametric Mann-Whitney-U-Test was performed using SPSS (IBM SPSS statistics 24, Chicago, IL, USA) to determine statistical evaluation of the FACS analyses, mass spectrometry, qRT-PCR, and blood plasma analyses. *P*-values p* ≤ 0.05 and p** ≤ 0.01 were considered to be statistically significant. Graphs were created using GraphPad Prism 5.0 and GraphPad Prism 7.0 (GraphPad Software, La Jolla, CA, USA). Data are reported as mean ± standard error of the mean (SEM).

## Results

### Spleen weight

Earlier research had shown a notable change of the liver- to–body weight ratio of *Npc1*^*−/−*^ mice after therapy [30]. Based on these findings, we further analysed the organ- to–body weight ratio of spleens (SW/BW). The evaluation of SW/BW ratio showed that sham-treated *Npc1*^*−/−*^ (*n* = 9) (0.08868 ± 0.02956) mice had an increased SW/BW ratio compared to sham-treated *Npc1*^*+/+*^ (*n* = 11) (0.05866 ± 0.01769) mice (Fig. 1b, *p* = 0.250). Both treated *Npc1*^*+/+*^ (*n* = 14) (0.11650 ± 0.03113) (Fig. 1b, *p* = 0.036) and *Npc1*^*−/−*^ (*n* = 12) (0.10660 ± 0.03078) mice (Fig. 1b, *p* = 0.006) had a significantly increased SW/BW ratio compared to sham-treated *Npc1*^*+/+*^ mice.

**Fig. 3:**
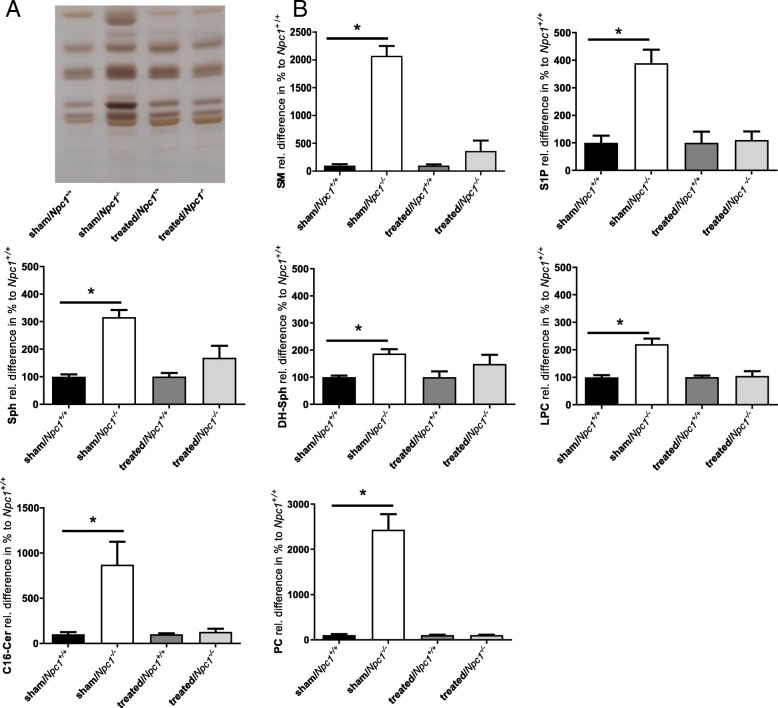
HPTLC-analysis (**a**) and mass spectroscopy (**b**) of spleen tissue of sham-treated and treated *Npc1*^*+/+*^ (sham, *n* = 3, treated, *n* = 3) mice. (**b**) Sham-treated *Npc1*^*+/+*^ and treated *Npc1*^*+/+*^ were set to 100%. Notice the band differences of sham-treated *Npc1*^*−/*−^ and the approximation to treated *Npc1*^*+/+*^ and *Npc1*^*−/−*^. Sham-treated *Npc1*^*−/−*^ showed a significant increase of SM, S1P, Sph, Dh-Sph, LPC, C16-Cer and PC. The treatment normalized the lipid levels. All data represent the mean ± SEM. p ≤ 0.05 was considered significant (*p ≤ 0.05). For *p*-values see text. SM: Sphingomyeline, S1P: Sphingosine-1-Phosphate, Sph: Sphingosine, DH-Sph: Dihydro-Sphingosine, C16-Cer: C16-Ceramide, LPC: Lysophosphatidylcholine, PC: Phosphatidylcholine

To identify possible reasons for increased spleen weight, we performed a lipid profile via HPTLC of sham-treated, treated *Npc1*^*+/+*^ mice, as well as sham-treated and treated *Npc1*^*−/−*^ mice (all groups *n* = 3). The band pattern of sham-treated or treated *Npc1*^*+/+*^ mice did not reveal clear differences. On the contrary, the lipid pattern of sham-treated *Npc1*^*−/−*^ mice showed noticeable band differences (Fig. 3a). The lipid sample of the treated *Npc1*^*−/−*^ approximated those of treated *Npc1*^*+/+*^ and sham-treated *Npc1*^*−/−*^ mice. To identify striking lipid bands of the HPTLC, we performed MS analyses (all groups *n* = 3). For data presentation, the values of treated and untreated *Npc1*^*+/+*^ were set to 100% and compared with the values of the respective *Npc1*^*−/−*^ mice. MS analyses demonstrate increased levels of S1P (388.77 ± 49.44; *p* = 0.05), sphingosine (Sph) (316.23 ± 26.14; *p* = 0.05), dihydro-sphingosine (DH-Sph) (186.90 ± 16.04; *p* = 0.05), lysophosphatidylcholine (LPC) (220.70 ± 19.79; *p* = 0.05), C16-ceramide (C16-Cer) (869.88 ± 256.05; *p* = 0.05), phosphatidylcholine (PC) (34:2) (2435.41 ± 342.05; *p* = 0.05), and sphingomyeline (SM) (2073.71 ± 178.34; *p* = 0.04) in spleens of *Npc1*^*−/−*^ mice compared to *Npc1*^*+/+*^ mice (S1P: 100.00 ± 26.51; Sph: 100.00 ± 8.34; DH-Sph: 100.00 ± 5.92; LPC: 100.00 ± 7.89; C16-Cer: 100.00 ± 25.4; PC: 100.00 ± 26.01; SM: 100.00 ± 27.01). The treatment of *Npc1*^*−/−*^ mice normalized the observed differences in sphingolipid and phospholipid profiles of SM (363.01 ± 185.18; *p* = 0.263), S1P (110.03 ± 31.31; *p* = 0.275), Sph (169.07 ± 43.15; *p* = 0.275), DH-Sph (148.82 ± 34.26;*p* = 0.275), LPC (104.85 ± 16.86; *p* = 0.827), C16-Cer (126.40 ± 36.69; *p* = 0.827), and PC (99.88 ± 11.05; *p* = 0.827) compared to treated *Npc1*^*+/+*^ mice (S1p: 100.00 ± 41.20; Sph: 100.00 ± 13.57; DH-Sph: 100.00 ± 21.75; LPC: 100.00 ± 6.08; C16-Cer: 100.00 ± 11.95; PC: 100.00 ± 11.01; SM: 100.00 ± 21.38) (Fig. 3b).

### S1P-receptors are differentially regulated

In order to identify the correlation between changes of phospholipids in spleen and lipid signalling we performed qRT-PCR of *S1pr1–5*. The ligand of all receptors is S1P that was strongly increased in sham-treated *Npc1*^*−/−*^ mice (Fig. 3). *S1pr1* was highly expressed in spleen, however, no significant changes between sham- or treated *Npc1*^*+/+*^ and *Npc1*^*−/−*^ mice were present. *S1pr2* was significantly increased in sham-treated *Npc1*^*−/−*^mice (0.0114 ± 0.0008) compared to *Npc1*^*+/+*^ mice (0.0051 ± 0.0006; *p* = 0.050). This increase was normalized after treatment (0.0050 ± 0.0003; *p* = 0.050). The treatment of *Npc1*^*+/+*^ mice (0.0053 ± 0.0003) had no effect compared to sham-treated *Npc1*^*+/+*^ mice (*p* = 0.827) and was significantly decreased compared to *Npc1*^*−/−*^ mice (*p* = 0.050). *S1pr3* was highly expressed in spleen but presented no significant differences between the different groups. *S1pr4* showed no significant changes in sham-treated *Npc1*^*−/−*^ mice (0.0903 ± 0.0084) compared to sham-treated *Npc1*^*+/+*^ mice (0.0715 ± 0.0063; *p* = 0.127). However, the treatment of *Npc1*^*+/+*^ mice (0.0548 ± 0.0003) led to a significant decrease of *S1pr4* compared to both sham-treated *Npc1*^*+/+*^ (*p* = 0.050) and sham-treated *Npc1*^*−/−*^ mice (*p* = 0.050). Treatment of *Npc1*^*−/−*^ mice (0.0635 ± 0.0103) showed a slight tendency of decreased *S1pr4* expression compared to both sham-treated *Npc1*^*+/+*^ (*p* = 0.0513) and *Npc1*^*−/−*^ mice (*p* = 0.127), though these were not significant. *S1pr5* was less expressed than all other *S1prs*. It revealed just a slight tendency of decreased expression in sham-treated *Npc1*^*−/−*^ mice (0.0007 ± 0.0001) compared to sham-treated *Npc1*^*+/+*^ mice (0.0013 ± 0.0002; *p* = 0.127). This was not normalized after treatment (0.0008 ± 0.0003). However, the treatment of *Npc1*^*+/+*^ mice (0.0007 ± 0.0001) revealed the tendency of decreased expression compared to sham-treated *Npc1*^*+/+*^ mice that was not significant.

### Functional histomorphology of the spleen

**Fig. 4:**
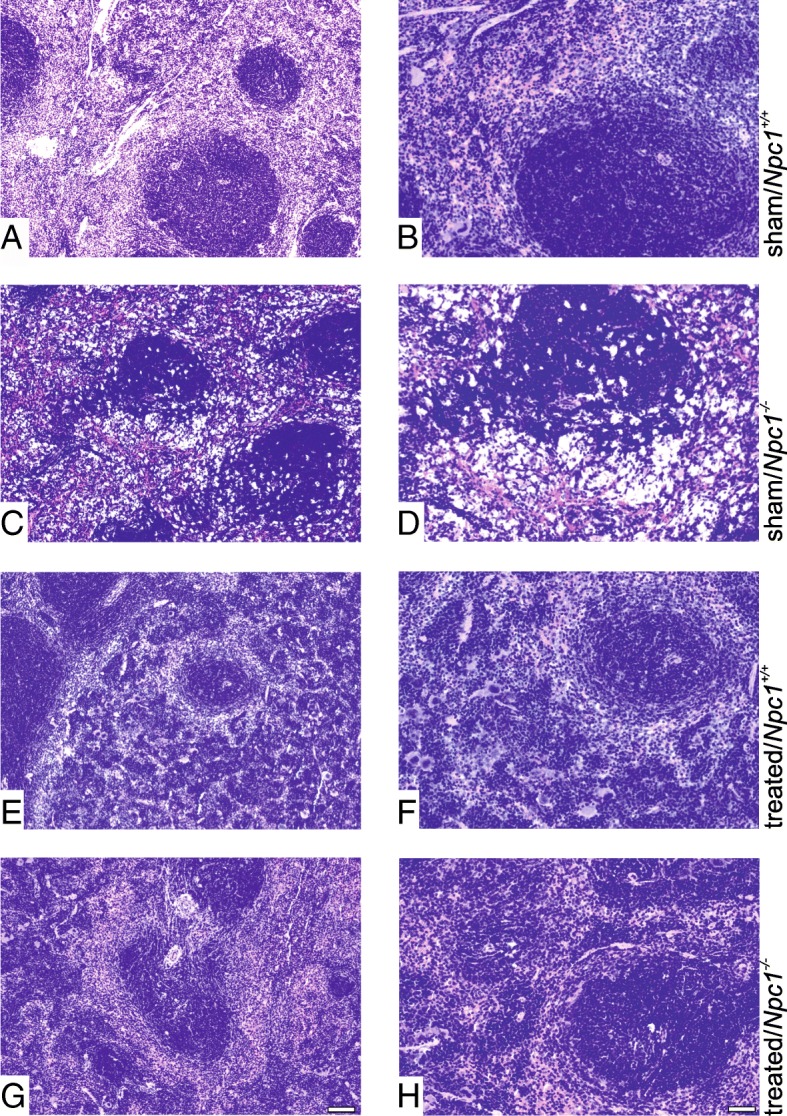
Hematoxylin & Eosin-stained images of spleen tissue of a sham-treated and treated *Npc1*^*+/+*^ (**a, e** and high magnification **a, f**) and of a sham-treated and treated *Npc1*^*−/−*^ mouse (**c, g** and high magnification **d**, **h**). Note the foam cells in the spleen tissue of a sham-treated *Npc1*^*−/−*^ mouse (**c**, **d**), and the amelioration in the spleen tissue of treated *Npc1*^*−/−*^ mouse (**g**, **h**). A scale bar is shown in **g**, which also applies to **a**,**c**,**e**: 100 μm and in **h**, also applies to **b**,**d**,**f**: 50 μm

Hematoxylin and eosin (H&E) staining of sham-treated or treated *Npc1*^*+/+*^ spleen showed normal morphology and regular lymph follicle architecture (Fig. 4a, b, e, f). In contrast, spleen tissue of sham-treated *Npc1*^*−/−*^ mice showed obvious morphological differences through the infiltration of foam cells, which conspicuously alter the spleen architecture by displacing the lymphoid follicles (Fig. 4c, d). Further, this phenomenon was remarkably reduced upon treatment. Treated *Npc1*^*−/−*^ mice revealed an amelioration of changes in spleen morphology, showing fewer foam cells, and generally resembled the *Npc1*^*+/+*^ phenotype (Fig. 4g, h).

### Treatment prevented inflammation in *Npc1*^*−/−*^

**Fig. 5:**
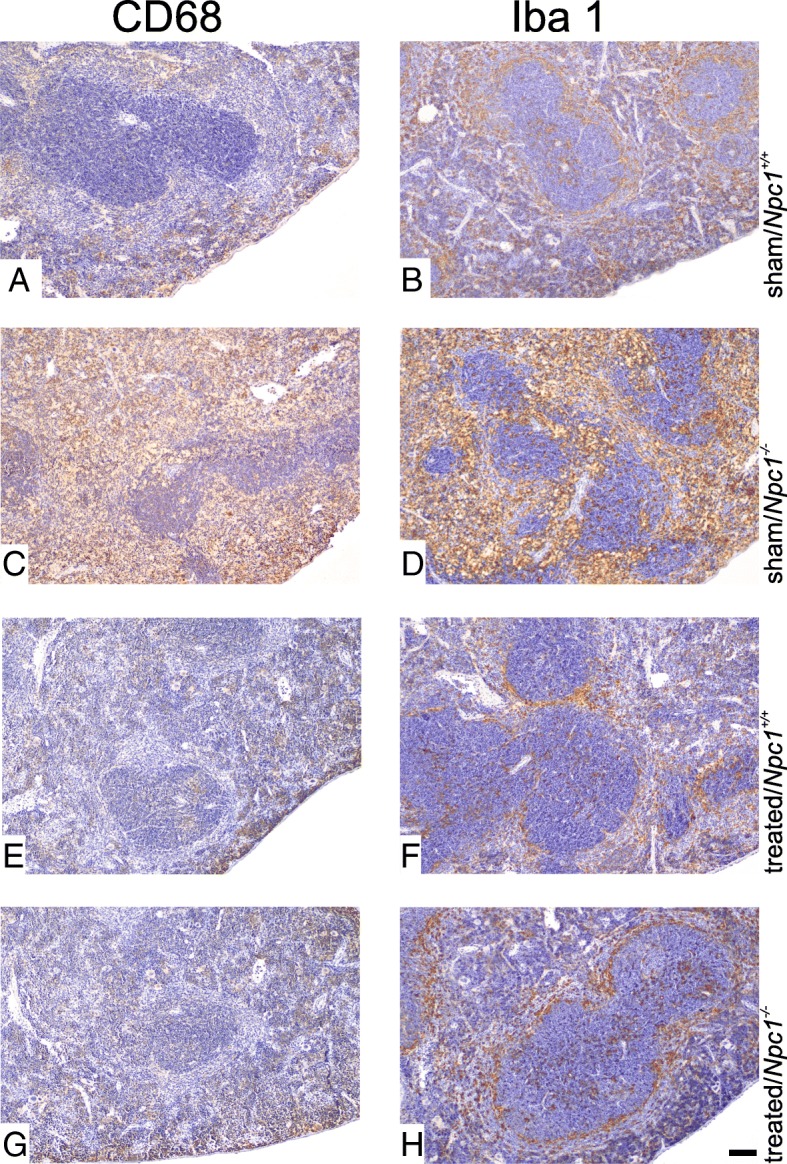
Paraffin sections from the spleen of sham-treated and treated *Npc1*^*+/+*^ and *Npc1*^*−/*−^ mice were immunhistochemically analyed with the macrophage markers CD68 (**a**, **c**, **e**, **g**) and Iba1 (**b**, **d**, **f**, **h**). The brown areas are positive reactions stained by DAB, counter stained with hematoxylin. Increased reaction of CD68 and Iba1 in sham-treated *Npc1*^*−/−*^ (**c**, **d**). The reaction of Iba1 is reduced in treated *Npc1*^*−/−*^ mouse (**h**). Scale bar: H: 50 μm and also applies to **a**-**g**

The cytochemical marker of macrophages CD68 and its mouse orthologue macrosialin is associated with inflammatory processes [45] and expressed in lysosomes of splenic macrophages. The immunohistochemistry of sham-treated *Npc1*^*+/+*^ showed regular accumulation and distribution of macrophages in the blood-containing red pulp (Fig. 5a). In sham-treated *Npc1*^*−/−*^ mice, the immunoreactivity was characterized by flooding of the red and white pulp with macrophages (Fig. 5c). In contrast, the immunohistochemical reaction of treated *Npc1*^*+/+*^ (Fig. 5e) and *Npc1*^*−/−*^ (Fig. 5g) mice was reduced and found to be similar to that of sham-treated *Npc1*^*+/+*^ mice.

Iba1, another marker of macrophages, has also been associated with inflammatory reactions and tissue repair [46]. In sham-treated *Npc1*^*−/−*^ mice, the number of Iba(+) cells in the spleen was markedly increased (Fig. 5d). However, sham-treated *Npc1*^*+/+*^ (Fig. 5b), treated *Npc1*^*+/+*^ (Fig. 5f) and treated *Npc1*^*−/−*^ animals (Fig. 5h) all showed similar and normal immunohistochemical reactions of Iba(+) cells in the spleen.

### Treatment induced alteration of immune cell numbers

**Fig. 6:**
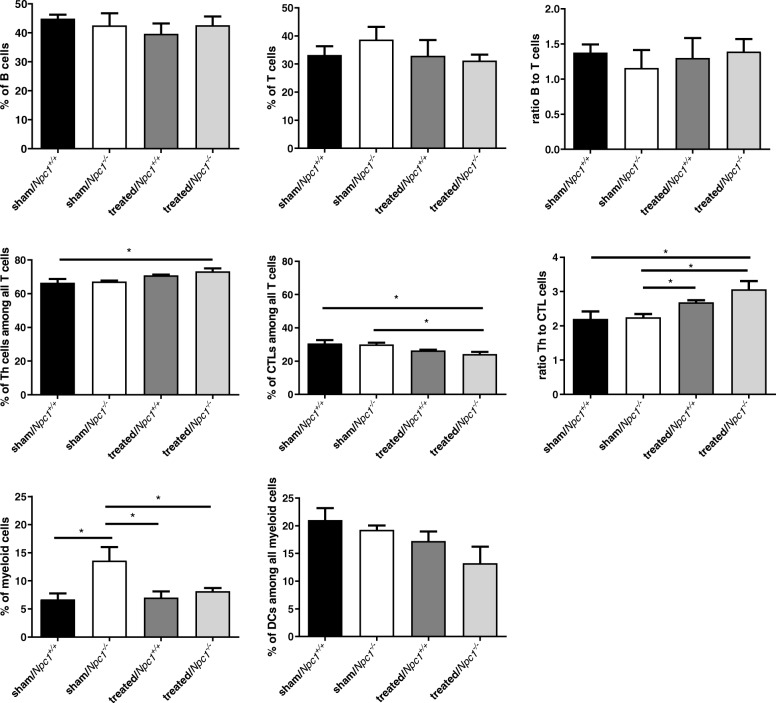
Quantitative analysis of leukocytes (*n* = 3) in spleen of sham-treated *Npc1*^*+/+*^ and *Npc1*^*−/−*^ mice as well as treated *Npc1*^*+/+*^ and treated *Npc1*^*−/−*^ mice. Sham-treated *Npc1*^*−/*−^ demonstrated significant accumulation of myeloid cells and slight changes of T cells and DCs. Treated *Npc1*^*−/−*^ showed a reduction of CTLs, myeloid cells and the rise of ratio Th to CTL cells. Note also the different changes of Th cells, CTLs and ratio between sham-treated *Npc1*^*+/+*^ and treated *Npc1*^*−/−*^. All data represent the mean ± SEM. *p* ≤ 0.05 was considered significant (*p ≤ 0.05). For p-values see text. Th: T helper cells, CTLs: Cytotoxic T lymphocytes, DCs: Dendritic cells

To investigate the protective activity and alteration of other immune cells on *Npc1*^*−/−*^and therapy, we performed FACS analyses of each of the 4 groups (*n* = 3, Fig. 6). The results revealed no significant alteration in B cells of sham-treated *Npc1*^*−/−*^ (42.57% ± 4.17%) compared to sham-treated *Npc1*^*+/+*^ (44.83% ± 1.39%; *p* = 0.513), treated *Npc1*^*+/+*^ (39.70 ±3.53%; *p* = 0.827), and treated *Npc1*^*−/−*^ (42.63% ± 3.04%; *p* = 0.827) mice. Likewise, the T cell values were not changed significantly. Nevertheless, treated *Npc1*^*+/+*^ (32.93% ± 5.64%; *p* = 0.513) and treated *Npc1*^*−/−*^ animals (31.17% ± 2.20%; *p* = 0.275) showed a slight decline compared to sham-treated *Npc1*^*+/+*^ (33.17% ± 3.20%; *p* = 0.275) and to sham-treated *Npc1*^*−/−*^ (38.67% ± 4.56%) mice. Furthermore, the B cell-to-T cell ratio showed no differences between *Npc1*^*+/+*^ (sham-treated: 1.37 ± 0.12; treated: 1.30 ± 0.28) or *Npc1*^*−/−*^ (sham-treated: 1.16 ± 0.26; treated: 1.39 ± 0.18) mice. However, the distribution of subclasses of T cells showed significant alterations. Here, the ratio T helper cells to T cells in sham-treated *Npc1*^*−/−*^ (67.20% ± 0.67%) and sham-treated *Npc1*^*+/+*^ (66.43% ± 2.28%; *p* = 0.827) mice was unchanged. In contrast, the number of T helper cells of treated *Npc1*^*+/+*^ (70.83% ± 0.44%) increased in contrast to sham-treated *Npc1*^*+/+*^ (*p* = 0.127) animals. Further, the number of T helper cells in treated *Npc1*^*−/−*^ mice (73.20% ± 1.76%) increased significantly compared with sham-treated *Npc1*^*+/+*^ mice (*p* = 0.050). Treated *Npc1*^*−/−*^ mice (24.13% ± 1.42%) had a significantly decreased number of cytotoxic T lymphocytes (CTLs) among all T cells, when compared to sham-treated *Npc1*^*+/+*^ (30.63% ± 2.08%; *p* = 0.050) and sham-treated *Npc1*^*−/−*^ mice (30.03% ± 1.04%; *p* = 0.050), but not to treated *Npc1*^*+/+*^ (26.43% ± 0.49%; *p* = 0.275) mice. The ratio CTLs to T cells in sham-treated *Npc1*^*−/−*^ and *Npc1*^*+/+*^ (*p* = 0.827) was slightly reduced. In addition, the CTLs number of treated *Npc1*^*+/+*^ mice was also, but not significantly, declined, compared to sham-treated *Npc1*^*+/+*^ mice (*p* = 0.127). In comparison to sham-treated *Npc1*^*−/−*^ mice (2.24 ± 0.10), the ratio of T helper cells to CTL cells showed a significant increase of treated *Npc1*^*−/−*^ (3.06 ± 0.24; *p* = 0.050) and treated *Npc1*^*+/+*^ mice (2.68 ± 0.07; *p* = 0.050). In addition, the ratio between treated *Npc1*^*+/+*^ to sham-treated *Npc1*^*+/+*^ mice (2.20 ± 0.22; *p* = 0.050) was also significantly increased. Sham-treated *Npc1*^*−/−*^ animals (13.60% ± 2.40%) showed a significantly altered number of myeloid cells when compared to *Npc1*^*+/+*^ mice (6.70% ± 1.07%; *p* = 0.050). The treatment of *Npc1*^*+/+*^ (7.03% ± 1.11%) and *Npc1*^*−/−*^ (8.17% ± 0.57%) animals reduced the number of myeloid cells to the level of the healthy mice significantly (*p* = 0.050). The ratio of dendritic cells to all myeloid cells was not changed significantly, although sham-treated *Npc1*^−/−^ (19.27% ± 0.76%; *p* = 0.513), treated *Npc1*^*−/−*^ (13.23% ± 3.01%; *p* = 0.127) and treated *Npc1*^*+/+*^ (17.23% ± 1.74%; *p* = 0.275) mice led to a moderate decline compared to sham-treated *Npc1*^*+/+*^ (21.03% ± 2.14%). In summary, B cells and T cells were not significantly changed. However, T helper cells and CTLs such as myeloid cells showed significant differences through the therapy.

### Transmission electron microscopy (TEM)

**Fig. 7:**
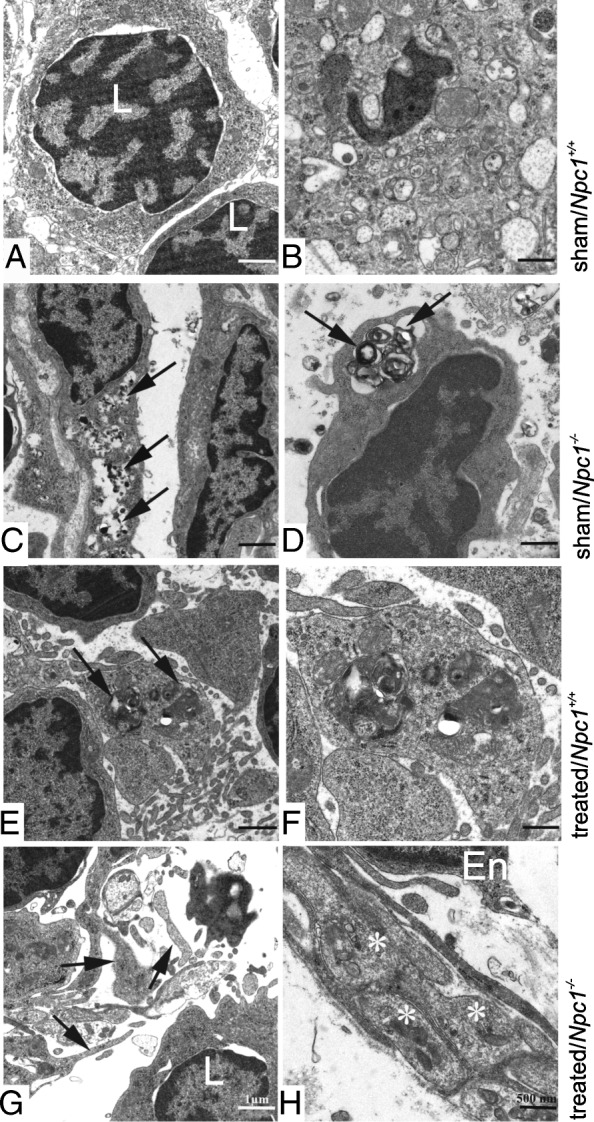
Transmission electron micrographs of splenic tissue. **a**, **b** Lymphocytes (L) and a macrophage (**b**) of *Npc1*^*+/+*^ mice. **c** myelin-like inclusions (arrows) in an endothelial cell of a sham-treated *Npc1*^*−/−*^ animal. Similar inclusions are seen in a dying sinusoidal cell (arrows, **d**). **e**, **f** congested material (arrows) in a sinusoidal macrophage in a treated *Npc1*^*−/−*^ animal. **g** sinusoidal reticulate cell processes (arrows) in a treated *Npc1*^*−/−*^ mouse, without pathologic inclusions. **h** sinus endothelial cell (En) and subendothelial cell processes (asterisks) of the red pulp without visible signs of pathologic inclusion material. Scale bars: 1 μm (left column); 500 nm (right column)

TEM of *Npc1*^*+/+*^ spleen tissue showed a heterogenous cell population, consisting of lymphocytes, macrophages, components of the reticular connective tissue, and sinusoid endothelium (Fig. 7a, b). In sham-treated *Npc1*^*−/−*^ animals, many cells contained typical myelin-like inclusions; especially the endothelial cells (Fig. 7c) and macrophages (Fig. 7d). These inclusions were absent in cells of treated *Npc1*^*+/+*^ mice (Fig. 7e, f) and almost absent in treated *Npc1*^*−/−*^ animals (Fig. 7h).

**Fig. 8:**
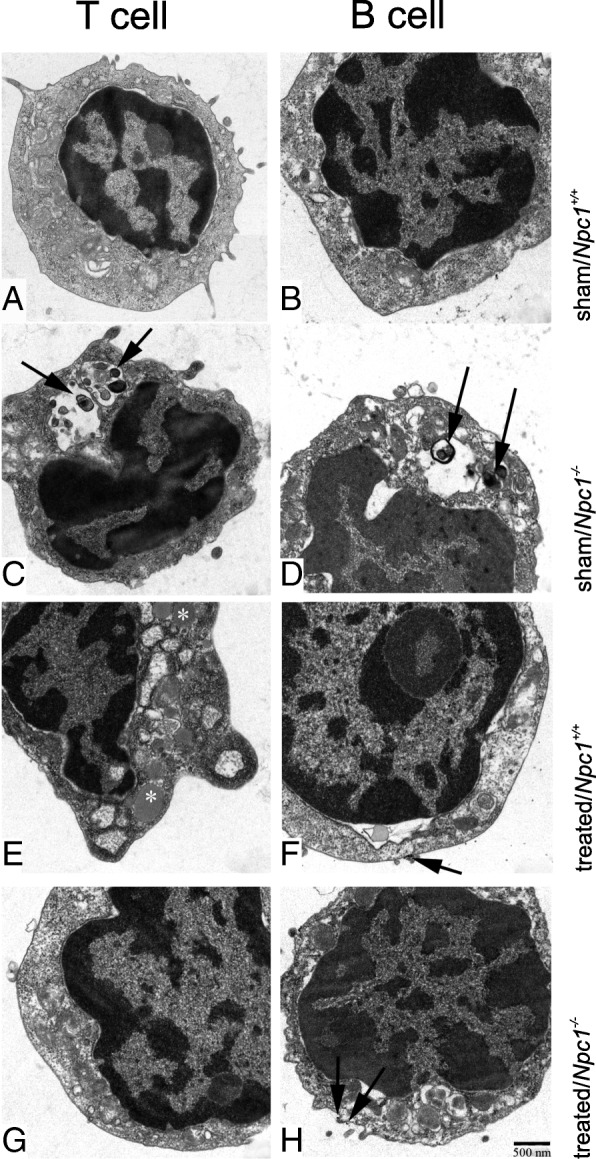
Transmission electron micrographs of FACS-sorted B- and T- lymphocytes. **a**, **b,** normal B- and T-lymphocytes. Only lymphocytes of sham-treated animals contain myelin-like inclusion material in enlarged cisterns (**c**, **d**, arrows). In **e**, lipid droplets are observed (asterisks). Arrows in (**f**) and (**h**) show microbeads used for isolating B cells from the lymphocyte suspension. Scale bar (in H): 500 nm

To examine whether subpopulations of lymphocytes were differently susceptible to NPC1 pathology, we sorted B and T cells by FACS and studied them ultrastructurally. Sham-treated T cells of *Npc1*^*+/+*^ animals exhibited extended endoplasmic reticulum, free ribosomes, and mitochondria (Fig. 8a). For comparison, B cells had fewer and less-developed organelles (Fig. 8b). Myelin-like inclusions in autophagosomes were seen in both T and B cells of *Npc1*^*−/−*^ sham-treated mice (Fig. 8c, d), which were not present in treated *Npc1*^*+/+*^ (Fig. 8e, f). Following treatment, no more lipid deposits were observed (Fig. 8g, h).

### Treatment prevents cellular redistribution of T cells and B cells

**Fig. 9:**
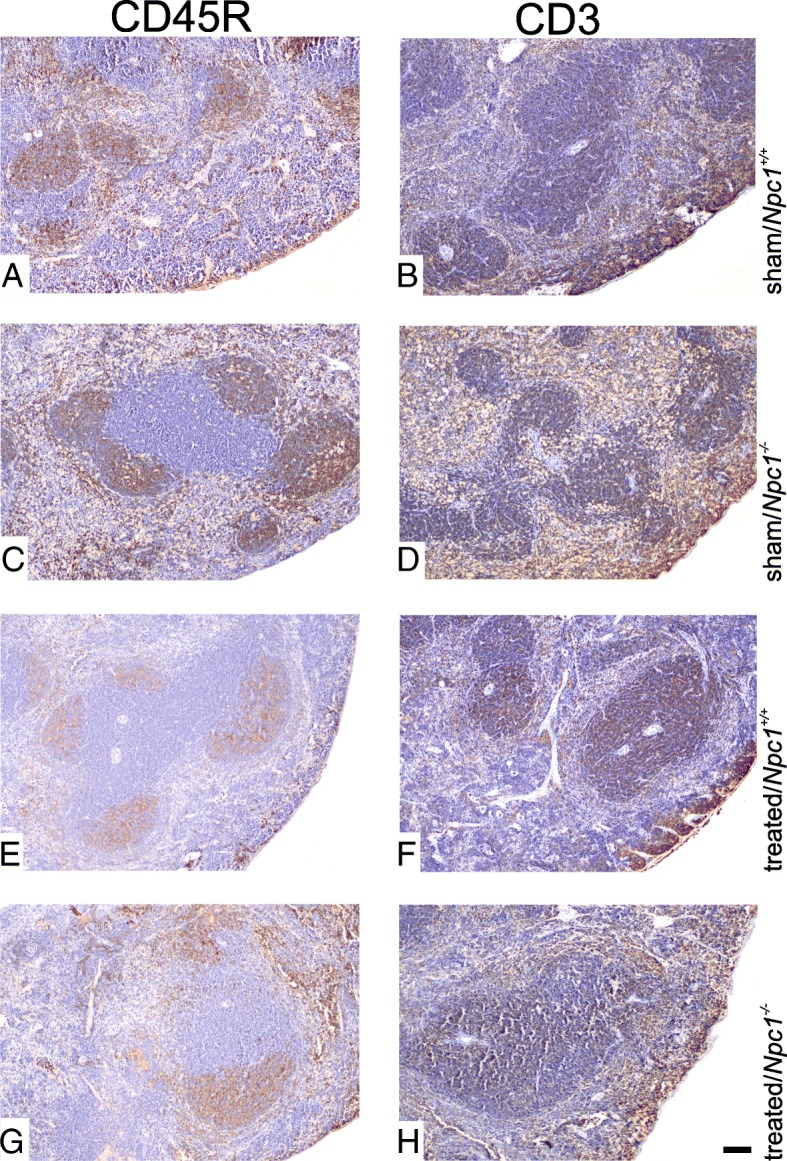
Paraffin sections from spleen of sham-treated and treated *Npc1*^*+/+*^ and *Npc1*^*−/−*^ mouse were immuniohistochemically analyzed with B cell marker CD45R (**a**, **c**, **e**, **g**) and T cell marker CD3 (**b**, **d**, **f**, **h**). The brown areas are positive reactions stained by DAB, counter stained with hematoxylin. Sham-treated *Npc1*^*−/−*^ demonstrated a different allocation of B cells (**c**) and T cells (**d**). This allocation was reversible after treatment. Scale bar: H: 50 μm and also applies to **a**-**g**

Based on the above-mentioned results of FACS analyses, we studied the distribution and alteration of the immune-specific markers CD45R and CD3. The CD45R marker represents B cell proliferation in the presence of T cells and is associated with activation of lymphocytes. The immunohistochemical data revealed, in addition to bloated pulp, that sham-treated *Npc1*^*−/−*^ (Fig. 9c) showed mild numbers of B cells in spleen sections when compared to treated *Npc1*^*−/−*^ mice (Fig. 9g) or to treated and sham-treated *Npc1*^*+/+*^ mice (Fig. 9a, e). We further performed CD3 immunoreactivity in spleen tissue. The immunoreactivity of the T cell marker CD3 revealed different numbers of CD3-positive cells in sham-treated *Npc1*^*−/−*^ mice compared to sham-treated *Npc1*^*+/+*^, treated *Npc1*^*+/+*^ and treated *Npc1*^*−/−*^ mice.

### No abnormal blood parameters in *Npc1*^*−/−*^ mice

**Fig. 10:**
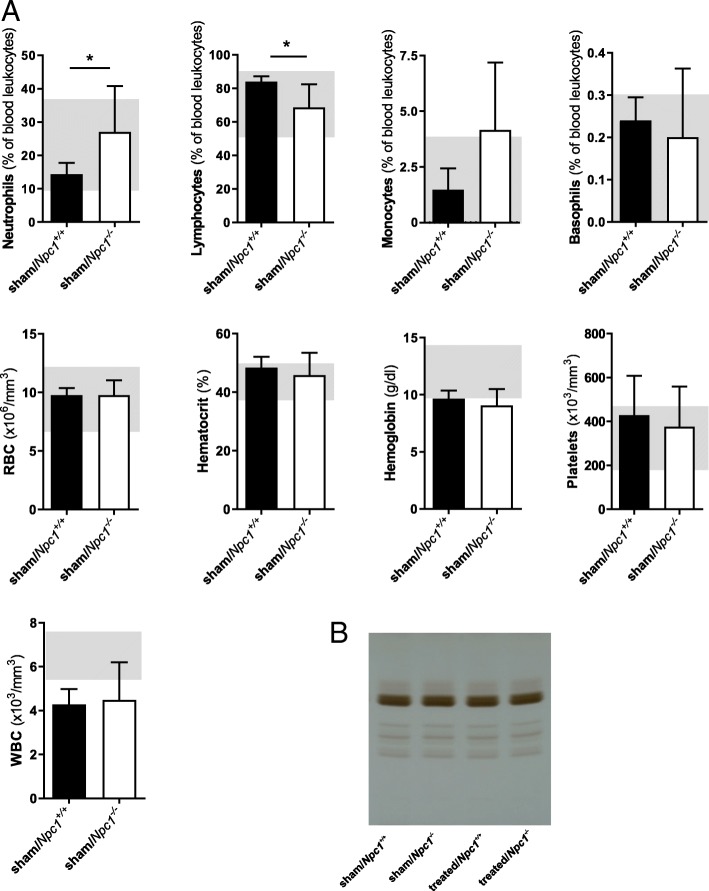
Analysis of whole blood (**a**) of sham-treated *Npc1*^*+/+*^ (*n* = 3) and *Npc1*^*−/−*^ (*n* = 3) mice. Reference values from the gray backside of “Handbook of Laboratory Animal - Management and Welfare”. Eosinophiles are non-existent in sham-treated *Npc1*^*+/+*^ and *Npc1*^*−/−*^. WBC, RBC, hematocrit, hemoglobin, platelets monocytes, and basophils showed no significant differences in both groups. Sham-treated *Npc1*^*−/−*^ demonstated significant changes of neutrophils and lymphoctes, but both results were in the reference values. HPTLC (high performance thin-layer chromatography)-image of blood plasma (**b**) of sham-treated and treated *Npc1*^*+/+*^ (sham, *n* = 3; treated, *n* = 3), and *Npc1*^*−/−*^ (sham, *n* = 3; treated, *n* = 3) mice. There were no obvious differences in any groups. Data are given as mean ± SEM; two-tailed nonparametic Mann-Whitney-U-Test; *p* ≤ 0.05 was considered significant (*p ≤ 0.05). RBC: red blood cells, WBC: white blood cells

In order to assess and exclude changes not only in lymphatic cells, but also in blood cells of sham-treated *Npc1*^*−/−*^ mice, we also explored blood analyses. First, we performed lipid profile analyses from blood serum of each group (*n* = 3) via HPTLC and semiquantitative HPTLC-analyses (data not shown). The analyses showed no band differences of the 4 groups (Fig. 10a). Second, we carried out bloodcount analyses (*n* = 3). The sham-treated *Npc1*^*−/−*^ mice exhibited a slight reduction of RBC (× 10^6^/mm^3^) (9.76 ± 1.29; *p* = 0.624), hematocrit (%) (45.78 ± 7.67; *p* = 0.624), hemoglobin (g/dl) (9.08 ± 1.43; *p* = 0.712), and platelets (× 10^3^/mm^3^) (376.50 ± 182.37; *p* = 0.624) compared to sham-treated *Npc1*^*+/+*^ mice (RBC: 9.75 ± 0.63; hematocrit: 48.38 ± 3.76; hemoglobin: 9.66 ± 0.70; platelets: 428.20 ± 179.21), which had previously been recognized [47]. Furthermore, there was no abnormality in basophils, WBC and monocytes detectable in sham-treated *Npc1*^*−/−*^ mice. The animals showed a mild decrease of basophils (%) (0.20 ± 0.16; *p* = 0.590), and a slight increase of WBC (× 10^3^/mm^3^) (4.48 ± 1.72; *p* = 0.624) and monocytes (%) (4.15 ± 3.03; *p* = 0.085) compared to *Npc1*^*+/+*^ mice (basophils: 0.24 ± 0.06; WBC: 4.28 ± 0.70; monocytes: 1.48 ± 0.96). The only significant noticeably different values were those of neutrophils (%) (27.03 ± 13.75; *p* = 0.027) and lymphocytes (%) (68.63 ± 13.8; *p* = 0.027) of sham-treated *Npc1*^*−/−*^ mice, compared to neutrophils (14.30 ± 3.45) and lymphocytes (83.98 ± 3.15) of sham-treated *Npc1*^*+/+*^ mice. Even though these parameters were statistically significantly different, the mean values are all in the reference area of “Handbook of Laboratory Animal – Management and Welfare”. With the exception of hemoglobin and WBC, all other values were in this reference area. No eosinophils were present in either group (Fig. 8b).

## Discussion

In the current study, we used different quantitative molecular- and cellular methods to provide insight into spleen alterations in NPC1 and the effects of the therapy. Our data confirm and extend previous results, also from other organs such as liver, namely, that treatment with miglustat/HPßCD/allopregnanolone in *Npc1*^*−/−*^ mice may prevent pathologic spleen morphology by reducing the number of myeloid cells and stabilizing lipid homeostasis. Interestingly, blood cell analysis remained unaltered in NPC1. However, we also showed for the first time that the treatment influences the number of cytotoxic T cells and T helper cells.

### Prevention of cellular and molecular changes in white pulpa areas

The spleen, the largest lymphoid organ in the body, is part of the mononuclear phagocyte system [48]. Beside blood filtering, storage and defense in the red pulpa, the spleen is responsible for the production for immune mediators, release of immunglobulins, storage and development for B and T lymphocytes [49]. Our results demonstrate the rise in spleen weight of untreated *Npc1*^*−/−*^ and treated *Npc1*^*−/−*^ and *Npc1*^*+/+*^ mice. In previous studies, an increase of spleen weight of untreated *Npc1*^*−/−*^ had already been shown due to extensively infiltrated foam cells [50], which is in agreement with our results. However, while weight increased during combination therapy, in particular by HPßCD [51], we report here an increase in the spleen/body weight ratio. This is in accordance with corresponding earlier findings in the liver [30]. In addition, we obtained an elevated number of CD68(+) and Iba 1(+) cells (macrophages) in spleen tissue of *Npc1*^*−/−*^ mice. This finding is also consistent with our observations in the olfactory bulb [52]. This massive infiltration of macrophages can be prevented by the treatment. Due to the progressive accumulation of lipids and other apparently toxic materials, followed by the impairment in degradation of autophagic substrates, cell death in *Npc1*^*−/−*^ mice is notoriously associated with inflammatory activity indicated by the infiltration of macrophages and/or microglia [30, 31, 52, 53]. Furthermore, the myelin-like inclusions, also named multilamellar inclusions, are also characteristic for NPC1. We identified these structures in different spleen cells of the red and white pulpa, indicating that the whole organ is affected. On the cellular level, we can distinguish between myeloid cells and lymphoid cells. Platt et al. and Speak et al. found an immune dysfunction with altered distribution and function of NK cells in *Npc1*^*−/−*^ mice, which belongs to the lymphoid cell population [19, 20]. In addition to these results, we demonstrated a different allocation of CD45 (B-cell marker) and CD3 (T-cell marker)- positive cells in *Npc1*^*−/−*^ spleen tissue. The numbers of these cells were not significantly altered. Interestingly, CTL and Th cells were significantly affected by the treatment.

### Metabolism of bioactive lipids is stabilized through the early therapeutic intervention in *Npc1*^*−/−*^ spleen tissue

The master enzymes sphingomyelinase and glucocerebrosidase are deficient in the absence of NPC1 function [54]. This results in an accumulation of different lipids in neural tissue and visceral organs. The abnormalities of sphingolipid metabolism in mutant NPC1 mice are already established [13, 4, 24]. In support of this, and in agreement with previous studies, we found that different sphingolipids and phospholipids accumulate in the spleen. In the present study, we observed an increase of SM, S1P, Sph, DH-Sph, LPC, C16-Cer, and PC, which coincides well with previous human- and mouse model studies [47, 54, 55]. Lipid homeostasis is a tightly regulated system. In particular, in lymphoid tissue such as the spleen, the balance between S1P on one hand, and ceramide and sphingosine on the other plays a critical role in determining whether a cell proliferates or dies [55]. Ceramide can be generated by hydrolysis of SM, and S1P is generated from sphingosine. However, early therapeutic intervention prevents the progressive accumulation of lipids in the *Npc1*^*−/−*^ spleen tissue. Interestingly, S1P gradients control egress of T and B cells from secondary lymphoid organs [56]. In this study, we showed for the first time that the number of Th cells is increased and that of CTL cells decreased in both treated, *Npc1*^*+/+*^ and *Npc1*^*−/−*^, animal groups. So far, it is not clear which one of the combined pharmacologic substances effects the T cell maturation or cell number. It has been suggested that both HPßCD and miglustat could have a general immunomodulatory effect [57–59]. Nevertheless, we show that the *S1p-receptor* gene expression is effected by *Npc1* mutation and by the treatment. A significant upregulation was found for the *S1pr2* in *Npc1*^−/−^, whereas the treatment prevents this increase in expression. It has been reported that S1P2 has an important regulatory impact on B cells. Moreover, the S1P2 expression on B cells can regulate the follicular positioning of B cells depending on S1P levels [60–62]. We identified a different allocation of CD45(+) cells in *Npc1*^*−/−*^ spleen tissue that may be due to the imbalance between S1P2 expression and S1P level. Interestingly, we observed a strongly elevated number of macrophages in *Npc1*^*−/−*^ spleen tissue. Furthermore, it has been suggested that S1P2 receptor expression reduces macrophage accumulation at sites of inflammation, whereas S1P is a regulator of macrophage recruitment to the site of inflammation [61, 63]. However, the treatment in *Npc1*^−/−^ animals prevents S1P2 overexpression and S1P increase in spleen tissue. In addition, we also identified a significant regulation of *S1pr4* in that we found a downregulation of this receptor in treated *Npc1*^*+/+*^ mice. *S1pr4* expression has been shown on T cells, suggesting that, beside other functions, S1PR4 may act on the migration of T cells towards S1P [64]. It could be speculated that the S1P4 receptor also influences the CTL and Th cell number in mice spleen after treatment. *Npc1*^*−/−*^ mice show a modified distribution and function of NK cells, which has also been shown in the S1P5 knock out mouse [19, 20]. We show that the mRNA expression of *S1pr5* is reduced in *Npc*1^−/−^ and treated animals. Although the results did not reach levels of significance, a clear tendency is detectable. Our results thus support the former findings.

### No remarkable changes in blood

In comparison to spleen abnormalities, the lipid profile of blood serum showed no differences in all 4 groups. The blood analysis revealed slight differences between healthy and mutant mice. The significant values in the cell count of lymphocytes and neutrophils are still in the reference area of the mouse model. In agreement with previous studies [8, 65], peripheral blood parameters were normal and there were no vacuolated peripheral blood lymphocytes while hepatosplenomegaly persists. However, Louwette et al. (2013) showed abnormal platelet formation and function in human NPC1 patients, while blood counts were normal [66]. This confirms that, basically, cell numbers in the blood and spleen are unchanged. There may, however, be a partial or total functional loss of individual cells through *Npc1* mutation and therapy.

### Side effects of combination treatment

In Europe only the substrate miglustat (Zavesca®, Actelion Pharmaceuticals, Allschwil, Switzerland) is an approved drug to treat humans with NPC1 disease [25]. In recent studies, HPßCD showed a potential therapeutic efficacy of escalating doses after lumbar intrathecal applications [67]. Both treatments are only used for symptomatic therapy in NPC1. In an earlier study, we observed that the monotherapy with HPßCD led to a reduction of hepatic lipids and to an amelioration of liver disease symptoms, but also to an increased cholesterol synthesis [30]. However, in our present study, we showed that the combination treatment decreases numbers of CTLs and increases that of Th cells. Whether this alteration in both T cell populations is a result of miglustat or HPßCD, or due to of the combination of both, has to be analyzed in future research. Nevertheless, new efforts were undertaken to identify better treatments. Sarah Spiegel’s group first showed evidence that FTY720/ fingolimod, so far used for treatment of multiple sclerosis, accumulates in the CNS when orally applied and is able to elevate NPC1 expression [68]. Therefore, FTY720 could be a potential new treatment for NPC1 patients, especially those with severe neurological sequelae.

## Conclusion

In summary, the *Npc1* mutation has a significant effect on entities of the red and white pulp of the spleen and leads to a redistribution of individual cell types and lipids. Preventive, continuous treatment with miglustat/HPßCD/allopregnanolone starting at birth almost completely preserves the splenic morphology. How far the function of specific immune cells after therapy is affected should be the subject of further research.

## Additional file

Additional file 1: **Table S1.** FAM-MGB coupled Taqman gene expression assays applied for qRT-PCR analyses of the spleen. (PDF 1357 kb)

## Data Availability

All data generated or analysed during this study are included in this published article and its supplementary information files.

## Abbreviations

ALLO: Allopregnanolone
CTLs: Cytotoxic T lymphocytes
DCs: Dendritic cells
HPßCD: 2-hydroxypropyl-ß-cyclodextrin
HPTLC: High-performance thin-layer chromatography
NPC1: Niemann-Pick type C1
*Npc1*^*−/−*^: NPC1 gene mutation
*Npc1*^*+/+*^: Wild-type without gene mutation
P: Postnatal day
S1P: Sphingosine-1-phosphate
*S1pr*: Sphingosine-1-phosphate receptor
Th: T helper cells
treated: Combination treatment with miglustat/HPßCD/allopregnanolone
