# Supplementary material for: A therapy with miglustat, 2-hydroxypropyl-ß-cyclodextrin and allopregnanolone restores splenic cholesterol homeostasis in Niemann-pick disease type C1
Source: Lipids Health Dis. 2019 Jun 28;18:146. doi: 10.1186/s12944-019-1088-2 (PMC6598286; doi:10.1186/s12944-019-1088-2)
Supplement: Supplementary file 1 — Table S1. FAM-MGB coupled Taqman gene expression assays applied for qRT-PCR analyses of the spleen. (PDF 1357 kb) [file 12944_2019_1088_MOESM1_ESM.pdf]

Gene	Assay ID/ primer and probe sequences
S1pr1, Sphingosine-1-phosphate receptor 1	Mm00514644_m1
S1pr2, Sphingosine-1-phosphate receptor 2	Accession-Nr.: NM_010333.4<br>Forward primer sequence (5'-3'): TTGGCTGATATCGCTGATTCTGG<br>Reverse primer sequence (5'-3'): TCCAGCTGGTTCAGACAATTCC<br>Reporter sequence (5'-3'): CAGGATGGGCAAGCCA
S1pr3, Sphingosine-1-phosphate receptor 3	Mm00515669_m1
S1pr4, Sphingosine-1-phosphate receptor 4	Mm00468695_s1
S1pr5, Sphingosine-1-phosphate receptor 5	Mm00474763_m1
Ppia, cyclophilin A	Mm02342430_g1
Actb, ß-Actin	Mm00607939_s1
